# Muscle Loss During First‐Line Chemotherapy Impairs Survival in Advanced Pancreatic Cancer Despite Adapted Physical Activity

**DOI:** 10.1002/jcsm.13595

**Published:** 2025-01-17

**Authors:** Pauline Parent, Frédéric Pigneur, Marc Hilmi, Aurélien Carnot, Marie‐Line Garcia Larnicol, Dewi Vernerey, Alain Luciani, Pascal Hammel, Julie Henriques, Cindy Neuzillet, Anthony Turpin

**Affiliations:** ^1^ Department of Medical Oncology Lille University Hospital Lille France; ^2^ University of Lille Lille France; ^3^ Department of Radiology, Henri Mondor Hospital, AP‐HP University of Paris Est, UPEC Créteil France; ^4^ Department of Medical Oncology, Institute Curie Université Versailles Saint‐Quentin, Paris Saclay Saint‐Cloud France; ^5^ GERCOR Paris France; ^6^ Department of Medical Oncology Centre Oscar Lambret Lille France; ^7^ Methodology and Quality of Life Unit in Oncology University of Besancon Besançon France; ^8^ Bourgogne Franche‐Comté University, INSERM, Etablissement Français du Sang Bourgogne Franche‐Comté, UMR1098, Interactions Hôte‐Greffon‐Tumeur/Ingénierie Cellulaire et Génique Besançon France; ^9^ Department of Digestive and Medical Oncology, Paul Brousse Hospital University Paris‐Saclay Villejuif France; ^10^ Lille University, CNRS INSERM UMR9020‐U1277, CANTHER Cancer Heterogeneity Plasticity and Resistance to Therapies Lille France

**Keywords:** adapted physical activity, CT scan, muscular mass loss, pancreatic cancer, quality of life, sarcopenia

## Abstract

**Background:**

Advanced pancreatic ductal adenocarcinoma (aPDAC) is often accompanied by significant muscle mass loss, contributing to poor prognosis. SarcAPACaP, an ancillary study of the GERCOR‐APACaP phase III trial, evaluated the role of adapted physical activity (APA) in aPDAC Western patients receiving first‐line chemotherapy. The study aimed to assess (1) the potential impact of computed tomography (CT)–quantified muscle mass before and during treatments on health‐related quality of life (HRQoL) and overall survival (OS) and (2) the role of APA in mitigating muscle mass loss.

**Methods:**

In the APACaP trial, aPDAC patients with ECOG performance status (PS) 0–2 were randomized 1:1 to usual care including first‐line chemotherapy or usual care plus a 16‐week home‐based APA program. In the SarcAPACaP study, the surface muscular index (SMI) was determined from L3 CT scan slices. Two patient populations were analysed: those with CT scan available at baseline (modified[m] intent‐to‐treat [ITT]1‐W0) and those with CT scans available at both W0 and W16 (mITT2 W0–W16). Low muscle mass was defined by low SMI with SMI < 41 cm^2^/m^2^ for women and < 43 and < 53 cm^2^/m^2^ for men with body max index < 25.0 and ≥ 25.0 kg/m^2^, respectively. Muscle loss was defined by the relative difference of SMI between W0 and W16 (100*[SMI W16–SMI W0]/SMI W0). In mITT2 W0–W16, patients were stratified into three groups based on the severity of muscle loss: none, moderate (0%–10%) and high (≥ 10%). Associations between muscle mass loss and OS, time until definitive deterioration (TUDD) of HRQoL and the effect of APA on loss of muscle mass were assessed.

**Results:**

Between October 2014 and May 2020, 313 patients were prospectively enrolled, with 225 in mITT1 W0 and 128 in mITT2 W0–W16, with 65 assigned to the APA arm. Both groups had similar baseline characteristics with comparable OS and TUDD. A low SMI at W0 was not associated with OS and TUDD of HRQoL in either group. Among mITT2 W0–W16 patients, high muscle mass loss (*n* = 27) independently predicted OS (*p* = 0.012) and showed a trend toward negatively affecting TUDD of HRQoL. Notably, APA did not mitigate muscle loss in our study population.

**Conclusions:**

Longitudinal muscle mass loss emerged as a predictive factor for both OS and HRQoL in aPDAC patients undergoing chemotherapy, while a low SMI at diagnosis did not provide prognostic value. APA did not impact muscle mass loss in this population.

## 1 Introduction

Pancreatic ductal adenocarcinoma (PDAC) is the seventh leading cause of cancer‐related mortality worldwide, with almost as many deaths (*n* = 466 000) as new cases (*n* = 496 000) according to GLOBOCAN 2020 estimates. Due to its increasing incidence and poor prognosis, PDAC is soon to become the second leading cause of cancer‐related death, second only to lung cancer [1, 2].

Advanced pancreatic ductal adenocarcinoma (aPDAC) is associated with high rates of muscle mass loss [3, 4]. Several factors contribute to the decreased muscle mass observed in PDAC patients, including reduced food intake, inflammatory and hypercatabolic syndrome, insulin resistance, pancreatic exocrine insufficiency and reduced physical activity [5, 6, 7]. Computed tomography (CT), commonly used for staging and treatment response monitoring, serves as a reliable tool for assessing muscle mass, incurring no additional patient burden or costs. Muscle mass quantification can be achieved through cross‐sectional imaging on CT scan at the middle of the third lumbar vertebra (L3) [8].

The incidence of sarcopenia in aPDAC patients ranges from 21% to 63% in different studies [9]. Recent meta‐analysis has suggested that low muscle mass at baseline is a poor prognostic factor in aPDAC patients [10, 11]. Additionally, an ancillary study of IMPACT trial of 94 aPDAC Western patients demonstrated a prognosis impact of longitudinal muscle mass analysis, particularly when loss exceeded 10% [12].

Physical activity, alongside nutritional intervention, stans as the main treatment against sarcopenia [13, 14]. The World Health Organization defines ‘physical activity’ as ‘any bodily movement produced by skeletal muscles that requires energy expenditure’. Adapted physical activity (APA) refers to physical activity or sports programs tailored specifically for frail individuals who, due to their physical, mental or social condition, are unable to participate in regular physical activity [15]. APA may be beneficial for patients with cancer by reducing disease or treatment‐related symptoms and improving physical fitness and muscle mass/strength. In the context of cancer, where patients are often symptomatic (fatigue, pain, …), APA involves the participation of professionals to support patients and personalize the intervention. Any physical activity, including APA, increases energy expenditure. This is why it must be combined with nutritional monitoring and intervention. APA does not mean that patients had minimal activity because they were frail. The APA program was adapted to the patient's physical condition.

Before APACaP study, only three studies have evaluated APA as a supportive care tool, mainly for localized PDAC patients before surgery [16, 17, 18]. The APACaP study, a prospective multicentric randomized open‐label phase III trial, recently evaluated the efficacy of APA in improving fatigue and health‐related quality of life (HRQoL) in patients with aPDAC [19]. In combination with standard care, APA improved the time to until definitive deterioration (TUDD) of several dimensions of HRQoL in aPDAC patients receiving first‐line chemotherapy. However, despite the association between low muscle mass and poor prognosis in these patients, the potential effect of APA on this parameter in patients receiving first line chemotherapy remains unclear.

The aim of the SarcAPACaP ancillary study of APACaP was to evaluate the potential impact of CT‐quantified muscle mass before and during first‐line chemotherapy on HRQoL and OS among aPDAC western patients. Additionally, the study evaluated the effect of APA on mitigating muscle mass loss.

## 2 Methods

### 2.1 Study Design

This is a retrospective study of patients included in the APACaP trial [19, 20]. The inclusion and exclusion criteria for the SarcAPACaP study were those of the APACaP trial with three additional exclusion criteria: (i) unavailable CT scan at baseline (W0), (ii) CT scan at week 0 (W0) performed more than 14 days before the start of the APA program and (iii) CT scan at week 16 (W16) performed either prior to week 12 or beyond week 20 after randomization.

Patients randomized to the experimental arm had a 16‐week APA program directed by an APA professional trainer, according to national and international guidelines [20, 21]. Patients in the standard arm received usual information and counselling by their caregivers about physical activity, according to the centre's routine practice and available scientific evidence.

The program combined (1) aerobic training tailored to each patient's preferences (walking, Nordic walking or cycling) with (2) resistance workouts using elastic bands (Appendix S1). Exercise duration, frequency and intensity were adapted to each patient by the APA monitor, according to standardized guidelines. HRQoL was registered monthly during the first 4 months, at 6 months and then every 3 months up to 2 years. Patients underwent CT scans at diagnosis/baseline (W0) and upon completion of the 16‐week program (W16). Routine clinical and biological follow‐up was conducted for up to 2 years post‐program completion.

### 2.2 Data Collection

The collected baseline characteristics included: clinical parameters (e.g., prior therapies, tumour stage, metastatic sites, ECOG performance status, pain, fatigue, anthropometric measurements [weight and height], pre‐diagnosis weight loss and height). For each patient, the initial physical activity level was estimated using the Global Physical Activity Questionnaire (GPAQ), which was used as a stratification parameter for randomization between experimental (APA) arm and standard arm. Baseline biological markers such as white blood cell count, haemoglobin, absolute neutrophil count, lactate dehydrogenase, C‐reactive protein and albumin were extracted from the APACaP case report forms.

### 2.3 Body Composition Measurements

**FIGURE 1:**
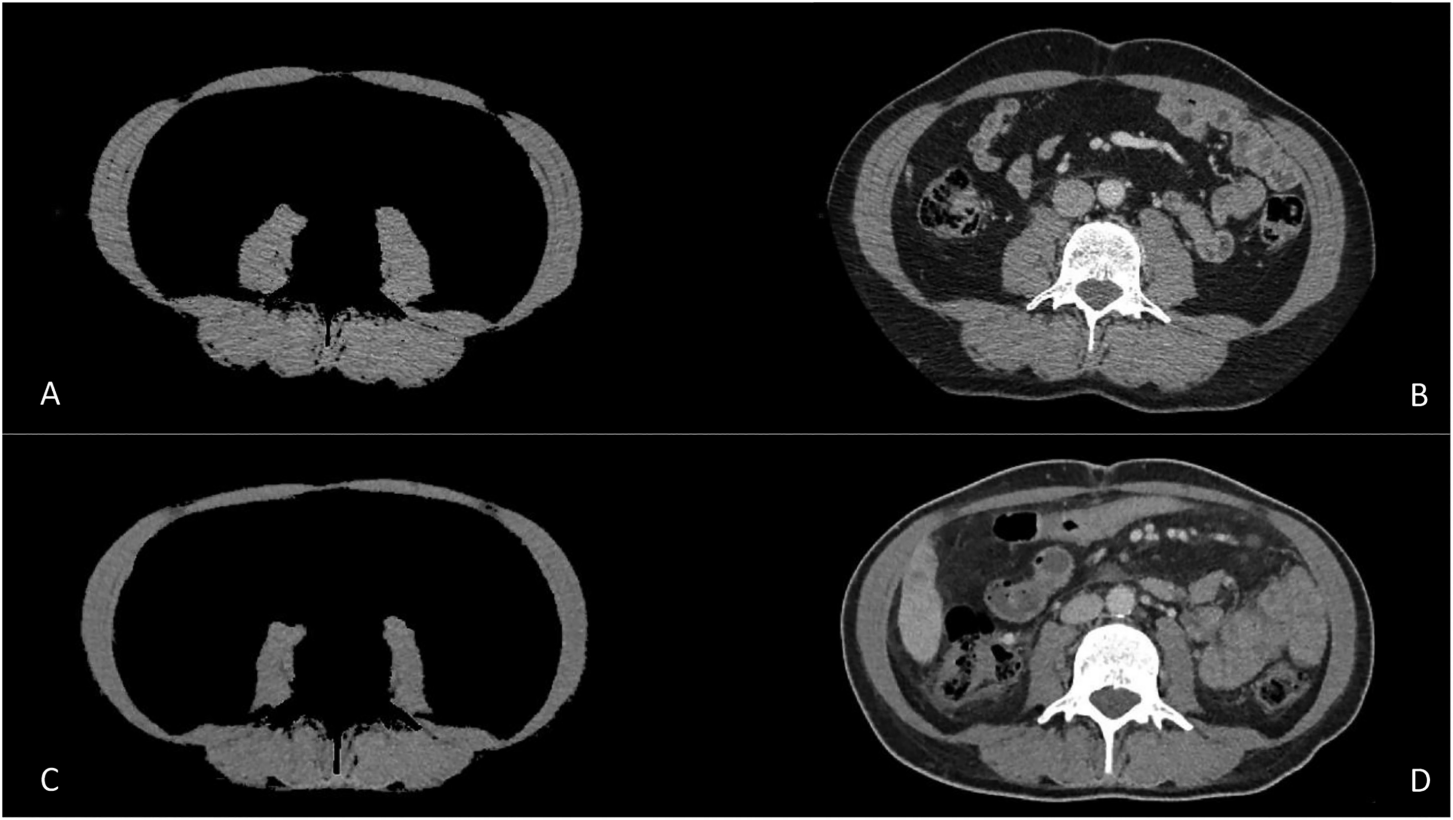
Two lumbar L3 CT scan slices at W0 (A and B) and at W16 (C and D) depict a male patient included in mITT2 population (APA arm), exhibiting a 20.6% decrease in muscle mass between W0 and W16. The patient main characteristics included an ECOG performance status of 0, moderately active GPAC score, a BMI of 24.9 kg/m^2^, the presences of liver metastasis, with SMI of 48.9 cm^2^/m^2^ at W0 and of 37.82 cm^2^/m^2^ at W16 SMI, OS of 4.5 months and TUDD of global health status of 0.82 months.

For each CT scan, two consecutive images extending from the middle of the 3rd lumbar vertebrae in the inferior direction were evaluated to determine the average of total lumbar muscle (psoas, erector spinae, quadratus lumborum, transversus abdominus, external and internal obliques and rectus abdominus) cross‐sectional area (CSA). SliceOmatic software (v5.0, Tomovision, Montreal, QC, Canada) was used to quantify CSA within predefined validated boundaries of −29 to +150 Hounsfield units. All CSAs were assessed by a trained doctor (PP). Concurrently, an expert radiologist (FP), also blind to all clinical data, reviewed the CT scans of 28 randomly selected patients to determine inter‐observer variability using a different dedicated workstation (Advantage Window v4.7; GE Healthcare, Buc, France). CT instrumentation parameters were identical for each patient at WO and W16 to optimize the reproducibility of CSA measurements. All CSAs were individually normalized for stature, as is conventional for body composition evaluation, resulting in SMI expressed in units of cm^2^/m^2^ (Figure 1) [8].

Patients with low muscle mass were identified based on low SMI using cut‐off values established by Martin et al. [22]. In summary, SMI < 41 cm^2^/m^2^ for women regardless of body mass index and < 43 and < 53 cm^2^/m^2^ for men with body max index < 25.0 and ≥ 25.0 kg/m^2^, respectively.

The monitoring of muscle mass between W0 and W16 was defined according to the following formula: 100*(SMI W16 − SMI W0)/SMI W0. Patients were divided into three groups based on SMI loss: no loss, moderate loss (< 10%) and high loss (≥ 10%).

### 2.4 Statistical Analyses

Two populations were analysed: (i) the modified intention‐to‐treat (mITT)1 W0 population corresponding to all randomized patients with SMI status available at diagnosis and (ii) the mITT2 W0–W16 population corresponding to patients from mITT1 W0 population with SMI available both at W0 and W16.

Baseline characteristics were described in the mITT1 W0 and mITT2 W0–W16 populations with median, minimum and maximum for quantitative variables and frequency and percentages for categorical variables. Patient characteristics were also described according to SMI status at W0 in the mITT W0 population and according to mass muscle loss between W16 and W0 in the mITT2 W0–W16 population.

HRQoL at baseline and W16 were compared between normal and low muscle mass using the Wilcoxon test and were assessed using the EORTC quality of life questionnaire (EORTC‐QLQ‐C30), targeting three dimensions: global health status (GHS), physical functioning (PF) and fatigue (FA). The TUDD of the HRQoL was defined as the time from the date of randomization to the first documented occurrence of deterioration defined as a decrease of at least 5 points over time compared to the baseline score, including death as an event in the absence of deterioration of HRQoL. Patients alive with no observed HRQoL deterioration were censored at the last available HRQoL assessment. Overall survival was defined as the time from randomization to death, with patients still alive being censored at the date of last follow‐up.

The Kaplan‐Meier method was used to estimate OS and TUDD, with curves compared between low and normal SMI and according to the evaluation of changes in muscle mass using the log‐rank test.

The proportion of patients with low and normal muscle mass was compared between arms of treatment separately at W0 and W16 with a chi‐square test. The evolution of total mass was compared between treatment arms using a Wilcoxon test.

The Pearson correlation for total of muscle mass at W0 and W16 between the two evaluators was estimated in 28 patients. The kappa coefficient was computed between the SMI status defined by the two evaluators (PP and FP) at W0 and W16 separately.

All statistical analyses were conducted using SAS Version 9.4 (SAS Institute, Cary NC) and R software version 4.1.1.

## 3 Results

### 3.1 Study Population

**FIGURE 2:**
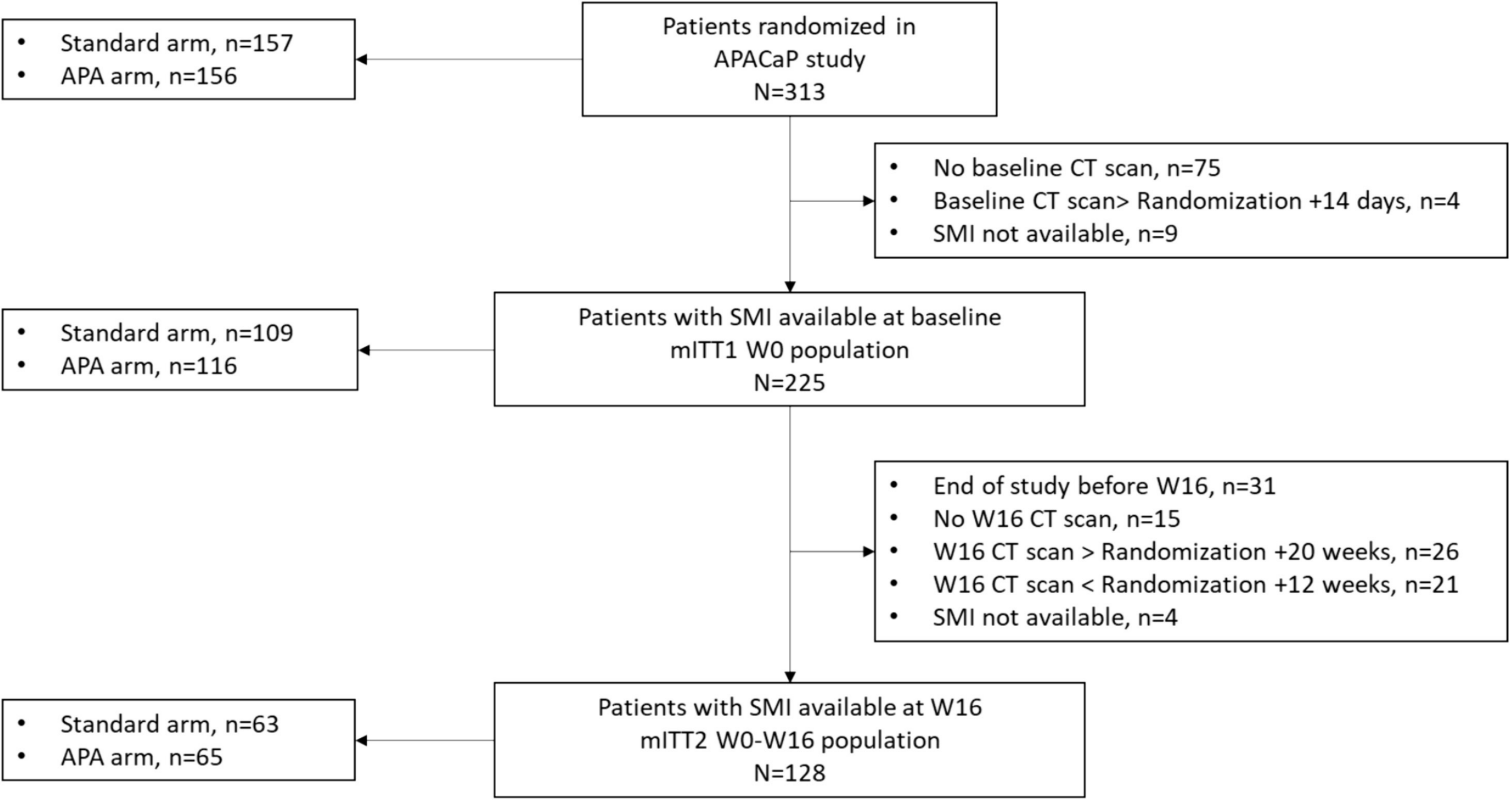
Flow‐chart of patient selection for the SarcAPACaP ancillary study.

A total of 225 patients in the mITT1 W0 population and 128 patients in the mITT2 W0–W16 population were included in this ancillary study. The detailed flowchart of the sample selection process is shown in Figure 2. Baseline characteristics were similar between patients in the APACaP and the SarcAPACaP study (Tables 1 and S1). Metastatic patients accounted for 94% and 96% of the mITT1 W0 and mITT2 W0–W16 populations, respectively, while 77% and 78% had an ECOS PS of 0–1. In both cohorts at baseline, patients were evenly distributed between very active, moderately active and inactive, according to GPAQ score.

**TABLE 1:** Baseline patient characteristics in the ancillary SarcAPACaP study compared to the APACaP population.

	APACaP population *N* = 313		SarcAPACaP population			
			mITT1 W0 *N* = 225		mITT2 W0–W16 *N* = 128	
	*n*	%	*n*	%	*n*	%
Sex, male	172	54.95	132	58.67	76	59.38
Age, years, median (min–max)	64 (29–87)		64 (29–87)		63 (29–87)	
Tumour stage at inclusion						
Metastatic	241	77.0	173	76.89	100	78.13
Locally advanced	72	23.00	52	23.11	28	21.88
ECOG PS						
0–1	292	93.29	211	93.78	123	96.09
2	21	6.71	14	6.22	5	3.91
GPAQ score						
Very active	111	35.46	81	36.00	44	34.38
Moderately active	96	30.67	71	31.56	45	35.16
Inactive	106	33.87	73	32.44	39	30.47
Chemotherapy regimen						
Weekly	35	11.18	25	11.11	11	8.59
Every 2 weeks	278	88.82	200	88.89	117	91.41
BMI, median (min–max)	23.8 (16–39.7)		23.6 (16.3–39.7)		24.2 (16.3–35.5)	
Albumin, g/L ≥ 35	227	81.36	161	80.50	92	83.64
CRP, g/L < 10	147	56.11	108	55.96	70	64.22
Median OS (95% CI)	14.7 (12.5–15.6)		15.3 (13.4–16.7)		17.1 (14.7–18.3)	
Median TUDD	11.1 (9.0–11.9)		11.6 (9.0–12.3)		11.9 (9.3–14.3)	

Abbreviations: BMI, body mass index; CRP, C‐reactive protein; ECOG PS, Eastern Cooperative Oncology Group performance status; GPAQ, Global Physical Activity Questionnaire; OS, overall survival; TUDD, time until definitive deterioration of quality of Life.

In mITT2 W0–W16, the evolution of muscle mass was correlated to weight loss with coefficient of correlation to 0.53 (Table S5). The median loss of SMI was more important in patients with progression disease at W16 (−6.23% [IQR = −15.18 to 0.99]) than in patients with controlled disease (−2.87% [IQR = −7.81 to 1.01]) but was not statistically significant (*p* = 0.2324).

The mean ratios of sessions performed during the weekly videocall compared with planned sessions in the mITT1 W0, mITT2 W0–W16, were, respectively, to 71.43% (STD: 37.11%) and 75.30% (STD: 35.36%). The percentages of patients who stopped the APA program prematurely were 23% and 10% in the mITT1 W0 and mITT2 W0–W16 populations, respectively.

The median OS was 17.1 months (95% CI: 14.7–18.3) and 15.3 months (95% CI: 13.4–16.7) in the mITT1 W0 and mITT2 W0–W16 populations, respectively, while the median TUDD was 11.6 months (95% CI: 9–12.3) and 11.9 months (95% CI: 9.3–14.3).

### 3.2 Radiologic Review

The SMI of CT scans of 28 patients at W0 and at W16 were reviewed, revealing a correlation between of 0.99751 and of 0.99651, respectively (Table S2).

### 3.3 The Impact of Low Muscle Mass on OS and HRQoL at W0 and W16

The description of patients based on normal and low SMI at W0 is presented in Table S3. In the mITT1 W0 population, 103 patients (46%) had a low SMI at diagnosis. Significant differences related to SMI at W0 were observed for sex and age: 59 (57%) patients with low muscle mass were female compared to 34 (28%) of patients with normal muscle mass (*p* < 0.001), with a median age of 66 years (Q1–Q3 : 58–72) for those with low muscle mass versus 62 years (Q1–Q3 : 56–68) for those with normal muscle mass (*p* = 0.005).

**FIGURE 3:**
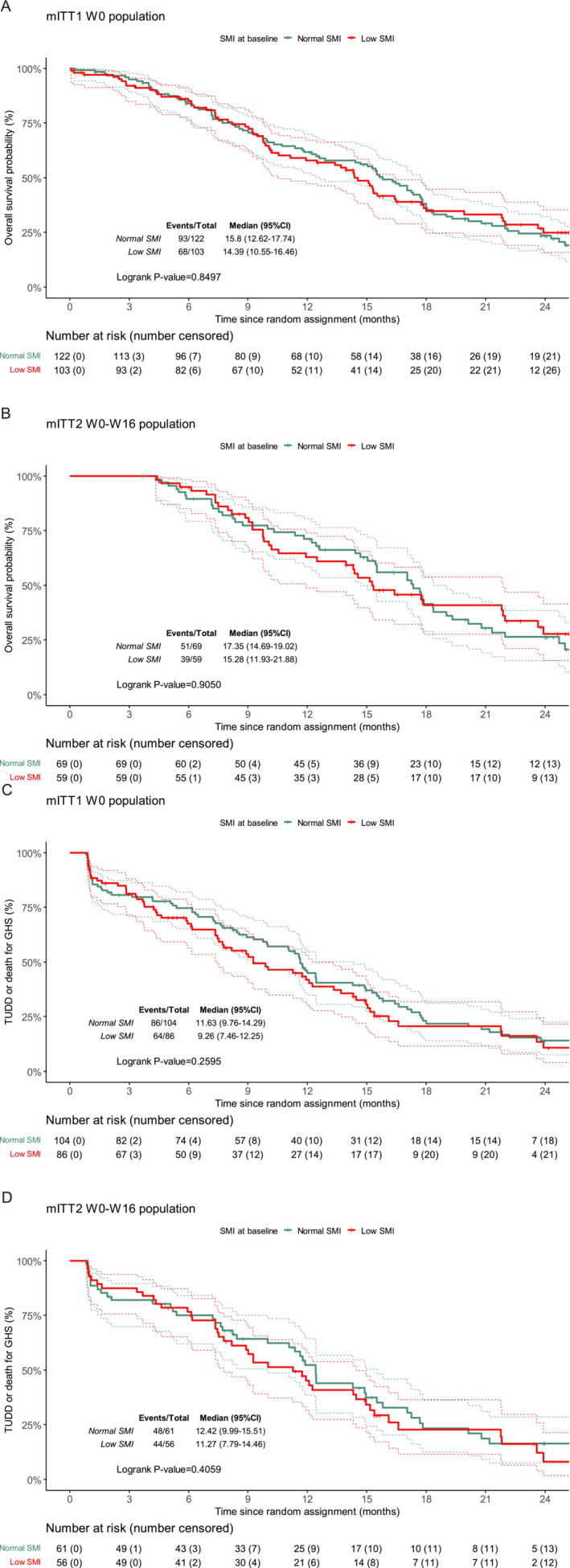
Overall survival (OS) and time until definitive deterioration of global health status (TUDD) according to the SMI status. OS in the mITT1 W0 (A) and mITT2 W0–W16 (B) populations. TUDD in the mITT1 W0 (C) and mITT2 W0–W16 (D) populations.

There was no difference observed in OS and TUDD for the three dimensions according to the muscle mass in the mITT1 W0 and mITT2 W0–W16 populations at W0 and at W16 (Figure 3 and Figure S1).

Unlike the EORTC QLQ‐C30 fatigue and GHS status dimensions, low muscle mass at W0 was associated with decreased PF when compared to patients with normal muscle mass (*p* = 0.012) (Table 2). In mITT2 W0–W16 population, patients with normal muscle mass at baseline had a median PF score of 88.9 points (SD 13.1), whereas those with low muscle mass scored 80.6 (SD 18.1) (*p* = 0017).

**TABLE 2:** Health‐related quality of life scores according to patient SMI status in the mITT1 and mITT2 populations of the SarcAPACaP study.

	mITT1 W0					mITT2 W0–W16				
	*N*	W0 Mean (SD)	*p*	W16 Mean (SD)	*p*	*N*	W0 Mean (SD)	*p*	W16 Mean (SD)	*p*
Global health status										
Normal SMI	73	62.7 (20.2)	0.26	70.0 (17.3)	0.316	44	62.1 (22.1)	0.28	70.5 (19.1)	0.2318
Low SMI	59	57.2 (22.7)		65.8 (19.0)		42	55.6 (23.2)		65.5 (18.1)	
Mean difference		5.5 (21.3)		4.2 (18.2)			6.6 (22.6)		5.0 (18.6)	
Fatigue										
Normal SMI	73	41.3 (25.6)	0.73	38.2 (21.5)	0.67	44	40.2 (26.7)	0.34	38.4 (24.7)	0.9360
Low SMI	59	44.2 (28.4)		37.3 (21.7)		42	47.9 (29.4)		37.8 (19.9)	
Mean difference		‐2.8 (26.9)		0.9 (21.6)			−7.7 (28.1)		0.6 (22.5)	
Physical functioning										
Normal SMI	73	89.4 (12.6)	**0.012**	84.8 (16.7)	0.313	44	88.9 (13.1)	**0.017**	86.5 (16.6) 80.0 (18.4)	**0.0405**
Low SMI	59	82.0 (18.2)		81.1 (20.4)		42	80.6 (18.1)		6.5 (17.5)	
Mean difference		7.4 (15.4)		3.7 (18.6)			8.3 (15.7)			

*Note:* Bold emphasis means that the result is statistically significant.

Abbreviations: W0, week 0; W16, week 16.

### 3.4 Impact of the Change of Muscle Mass Between W0 and W16 on OS and HRQoL

**FIGURE 4:**
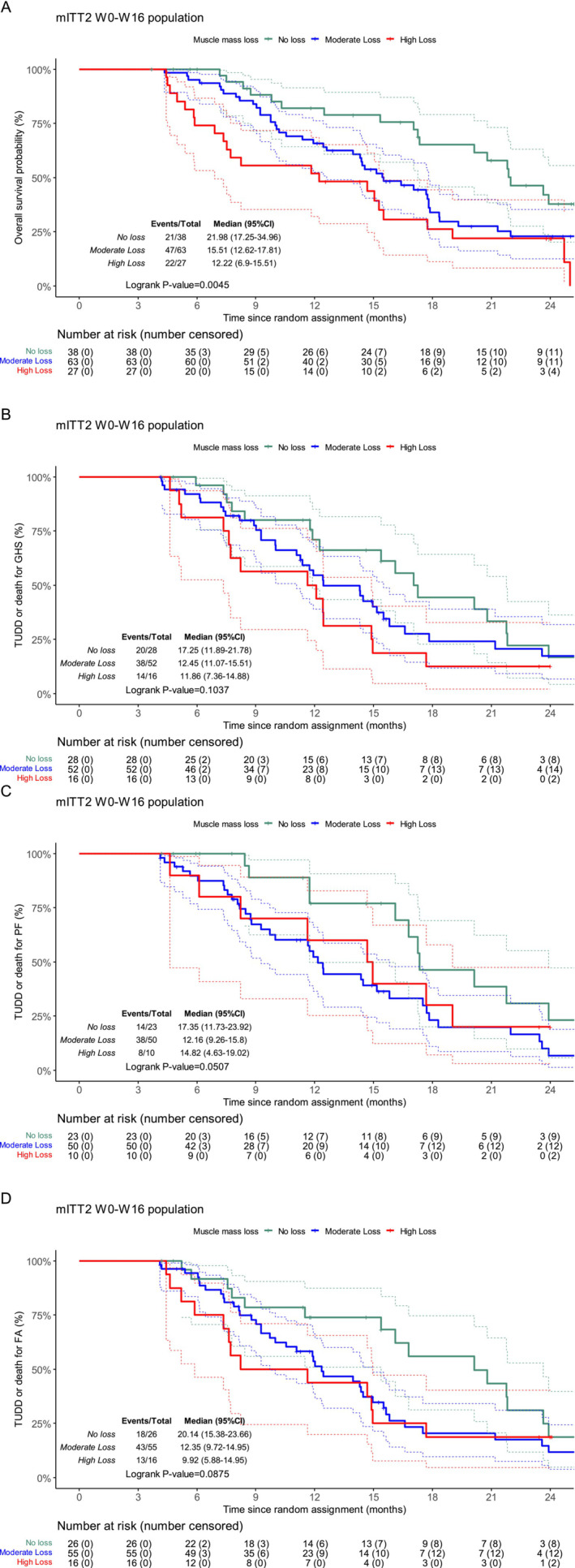
Overall survival according to evolution of muscle mass between W0 and W16 in the mITT2 W0–W16 population (A). Time until definitive deterioration of global health status (B), physical functioning (C) and fatigue (D) according to evolution of muscle mass between W0 and W16 in the mITT2 W0–W16 population.

The patient characteristics based on to the change in muscle mass are summarized in Table S4. In the mITT2 W0–W16 population, a statistically significant difference in OS was observed based on the change in muscle mass between W0 and W16 (*p* = 0.005). Patients who experienced no loss of muscle mass had a better median OS than those who had muscle mass loss (22.0 months [95% CI: 17.2–35.0] vs. 12.2 months [95% CI: 6.9–15.5]) (Figure 4).

The change in muscle mass appeared to be associated with TUDD of the HRQoL across the three dimensions (Figure 4), and there was a statistically non‐significant trend. Concerning the GHS dimension, the median TUDD for no loss, moderate loss and high loss was 17.2 months (95% CI: 11.9–21.8), 12.5 months (95% CI: 11.1–15.5) and 11.9 months (95% CI: 7.4–14.9), respectively (*p* = 0.1037). For the PF dimension, the median TUDD for no loss, moderate loss and high loss was of 17.3 months (95% CI: 11.7–23.9), 12.2 months (95% CI: 9.3–15.8) and 14.8 months (95% CI: 4.6–19.0), respectively (*p* = 0.0507). For the fatigue dimension, the median TUDD for no loss, moderate loss and high loss was 20.1 months (95% CI: 15.4–23.7), 12.4 months (95% CI: 9.7–14.9) and 9.9 months (95% CI: 5.9–14.9), respectively (*p* = 0.0875).

### 3.5 The Impact of APA on Muscle Mass

Patients with normal and low SMI were not evenly distributed across the two arms: A higher proportion of patients in the standard arm had a low SMI (56%, *n* = 58) compared to those in the experimental arm (44%, *n* = 45; *p* = 0.03, Table S2). Between W0 and W16, patients experienced an average muscle mass loss with a median relative reduction in muscle mass of −2.97% (Q1–Q3 : −9.0 to 1.3). The total muscle mass loss between W0 and W16 did not differ significantly between the treatment arms: −2.87% (Q1–Q3 : −7.8 to 1.0) for the APA arm versus −3.55% (Q1–Q3 : −9.0 to 1.6) for the standard arm (*p* = 0.89).

Finally, in APA patients with good compliance (more than 75% of sessions completed), the total loss of muscle mass between W0 and W16 was lower at −2.39% (Q1–Q3 : −7.23 to 0.94) than in patients with ‘poor’ compliance, with a total loss of muscle mass of −8.76% (Q1–Q3 : −19.08 to 3.10) with no significantly *p* value (*p* = 0.17).

**FIGURE 5:**
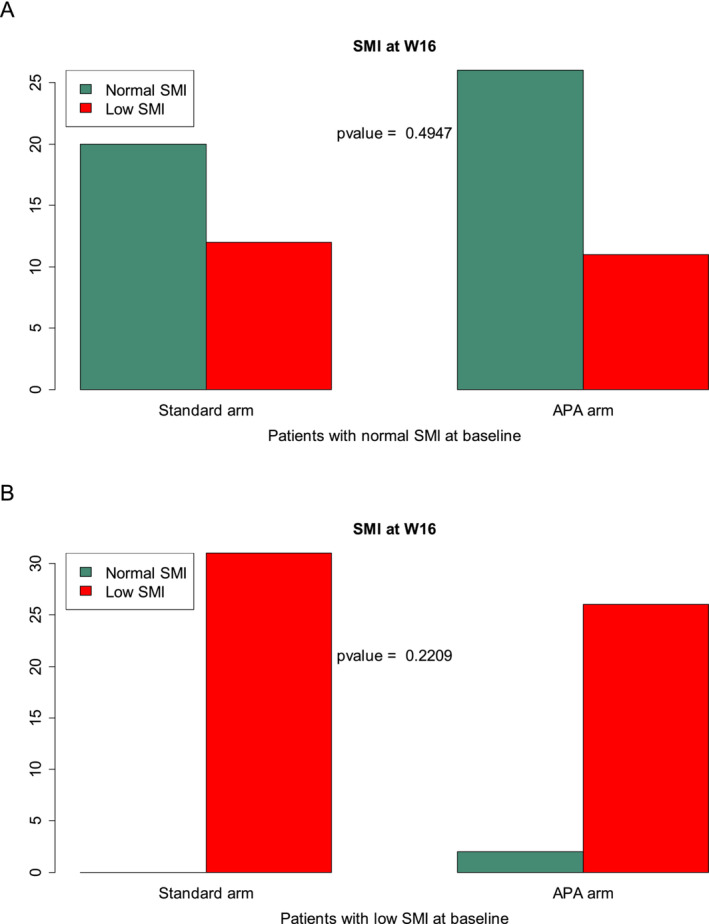
Evolution of SMI at W16 in patients with normal SMI at W0 according to treatment arm (A). Evolution of SMI at W16 in patients with low SMI at W0 according to treatment arm (B).

**FIGURE 6:**
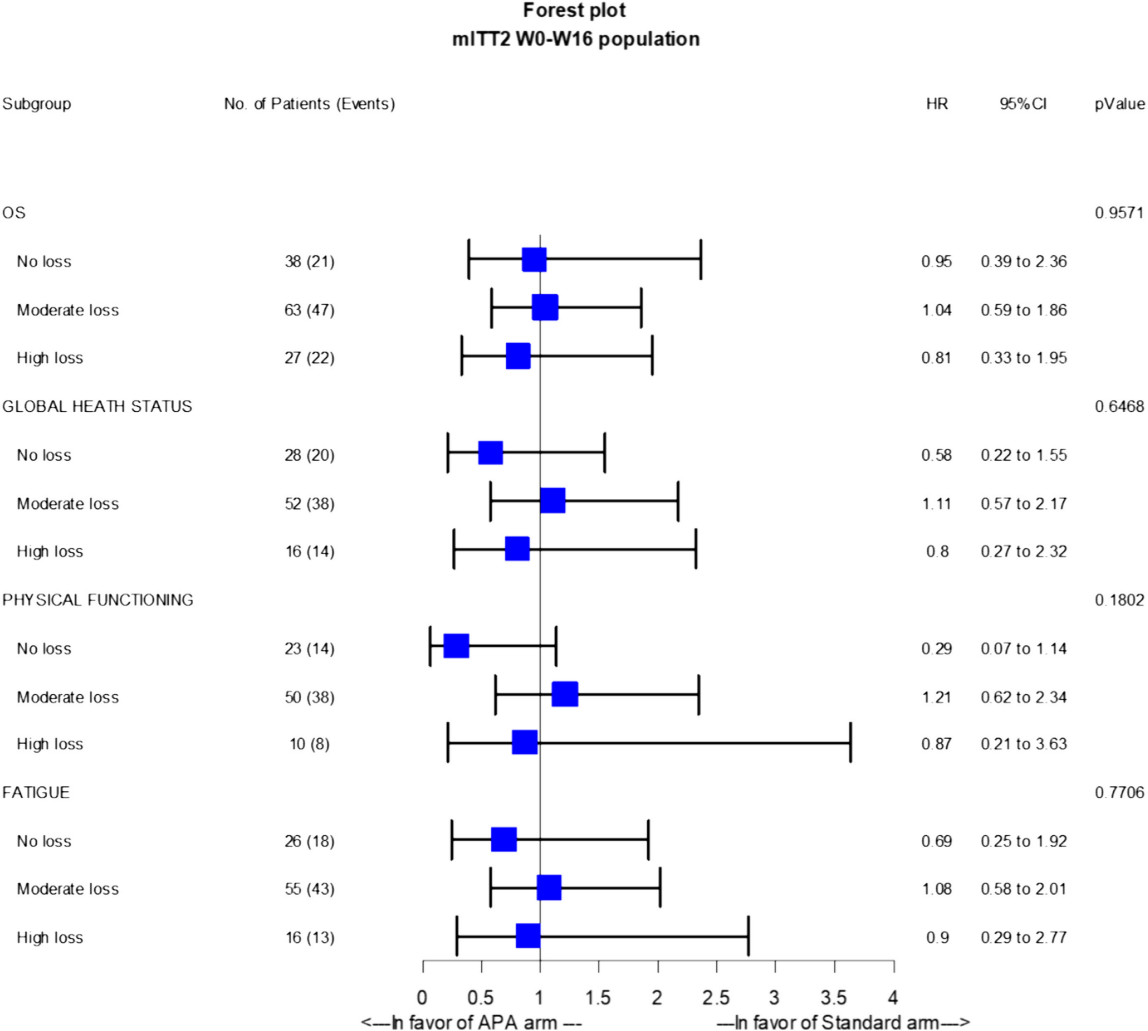
Forest plot of the impact of APA on OS and HRQoL according to muscle mass evolution between W0 and W16.

We analysed the effect of APA intervention on muscle mass loss according to initial SMI class. Among patients with normal SMI at W0, the proportion with low SMI at W16 did not significantly differ by treatment arms: 37.5% versus 29.73% (*p* = 0.49) in the standard and APA arms, respectively. In patients with low SMI at W0, nearly all patients maintained low SMI regardless of treatment arm (*p* > 0.05; Figure 5). There was no statistically significant interaction between treatment arm and muscle mass loss for OS or TUDD (*p* > 0.05; Figure 6).

Finally, in patients with good compliance (more than 75% of completed sessions), the relative difference of SMI between W0 and W16 was lower to −2.39 (Q1–Q3 : −7.23 to 0.94).

## 4 Discussion

We present the results of SarcAPACAP, an ancillary study derived from the APACaP phase III trial, which is the first to evaluate the association between HRQoL and muscle mass loss in a large prospective cohort of aPDAC patients receiving homogeneous first‐line chemotherapy. Moreover, the impact of APA, on muscle mass as non‐pharmacological intervention associated to nutrition in a randomized clinical trial, brings new value.

Contrary to low muscle mass (low SMI), the evolution of muscle mass impacted outcomes mPDAC patients in a prospective randomized phase 3 clinical trial. Our analysis revealed no significant impact of a low SMI on OS and HRQoL in aPDAC patients treated with chemotherapy at W0 and W16. This aligns with findings showing the lack of prognostic impact of low SMI at diagnosis in colorectal cancer patients [23]. A recent retrospective study by Lee et al. reported a significant correlation between SMI was and OS in male Asian patients (HR = 0.59; 95% CI: 0.43–0.80; *p* < 0.001), with no such association found in females [4]. However, differences in anthropometric measurements and thresholds for low muscle mass between Asian and European populations warrant caution in interpreting these findings.

Furthermore, in our prospective study, we found that that a loss of muscle mass ≥10% between W0 and W16 was an independent factor associated with poor OS and tended to be associated with TUDD of the HRQoL across three targeted dimensions of EORTC QLQ‐C30 questionnaire (fatigue, PF and GHS). This tendency is possibly linked to a lack of power. This finding is consistent with the results of the IMPACT study of 94 PDAC patients, which showed that an early loss of muscle mass ≥ 10%, measured at the first radiological evaluation and compared with baseline, influences prognosis [12]. Our results provide external validation for the 10% cut‐off used in the IMPACT study, thereby reinforcing its value in predicting patient outcomes. Lee et al. also reported a significant decreased in SMI between the initial and 2‐month follow‐up [4], underscoring the importance of early longitudinal analysis of SMI. Early assessment of muscle mass loss better reflects tumour changes and sensitivity to treatment, both major factors influencing OS and HRQoL in poor‐prognosis cancers [5].

Regarding the prevalence of low SMI at diagnosis (46%), our study aligns with prior studies using Martin's criteria, which have reported rates ranging from 21% to 63% [8, 9, 21, 24]. However, in the IMPACT study, a higher baseline sarcopenia rate (73%) was reported, albeit defined by different criteria (Prado) [12, 25].

In SarcAPACaP, APA did not significantly reduce the occurrence of low SMI in patients with normal baseline SMI nor reverse low muscle mass in aPDAC patients with baseline low SMI, after 16 weeks of APA intervention concomitant to systemic chemotherapy. This suggests that the APA program proposed in our study may insufficiently prevent the onset of loss of muscle mass in this population, given its multifactorial and irreversible nature in PDAC patients, with the tumour evolution under treatment as a powerful driver of muscle loss. Several explanations may have compromised the interpretation of the effect of APA on muscle mass in our study. Here, we have information about the mode of exercise in APA, not concerning certain aspects, such as volume, intensity, or progression of APA. Without monitoring of APA implementation and its effects on physical fitness, we cannot conclude definitively on the real influence of APA on muscle mass. But analyses are ongoing on physical performance measures such as muscle strength, cardiovascular fitness or physical function. Second, the initial population was not stratified for loss of muscle mass, resulting in an imbalance favouring the APA arm; for reason of feasibility (availability of the results for patient stratification of randomization), we used GPAQ as a surrogate of patient physical condition [19]. Third, the proposed exercises may not have been intensive enough or optimal to target L3 muscle, while the nutritional support (which was based on adherence to national guidelines but not standardized with mandatory goals for protein and calory intakes) may have been insufficient. Last, the CT scan–based muscle quantification at L3 may not be the best tool for evaluating the impact of APA [26]. These results align with the results of the PancFit study in localized PDAC patients undergoing neoadjuvant treatment, which similarly found no discernible effect of APA on physical function and HRQoL than aPDAC patients. Unlike aPDAC, the pathophysiology in the perioperative situation is different: acute stress would require more intensive preadaptation with intense exercises [27]. Nevertheless, both in vitro and clinical evidence suggests that APA, when combined with a nutritional intervention strategy, could mitigate or even prevent muscle mass loss [6]. Moreover, a robust pre‐clinical rationale posits a direct link between chemotherapy (platinum‐based regimens) and muscle mass reduction [28, 29]. A literature review from 2019 further hints at chemotherapy toxicity as potential contributor to low muscle mass [30]. The efficacy of chemotherapy improves tumoral control and reduces hypercatabolism [31].

According to ‘SCAN’ study, clinicians could not evaluate muscle mass in consultation [32]. With the advent of AI in a near future, the measurement of muscle mass and muscle mass loss will become more systematic. Noted below the RECIST 1.1 evaluation, this systematic assessment will need to be validated as a tool to adapt therapeutic intervention according to muscular state and metabolic level and could impact toxicities gestion of chemotherapy.

Several active phase III trials (NCT02330926, NCT04136249) are investigating the efficacy of a multimodal strategy for the general physical wasting and malnutrition syndrome, cachexia. This strategy combines drugs targeting inflammatory or other cachectic factors (such as interleukin 6, GDF15) with APA and nutritional intake, based on the results of previous studies [33, 34]. However, a recent review by Grande et al. concluded that there is a lack of efficacy, acceptability and safety of APA in adults with cachexia [35].

In our study, despite good concordance of muscle mass measures between the two observers, there are a few limitations. First, not all patients of the APACaP study were included due to a lack of available imaging data. Additionally, technical issues such as the timing of CT injection, slice thickness and the intrinsic qualities of the CT material need clarification, as they may influence longitudinal CSA measurements [36, 37, 38]. We have attempted to limit these biases by considering portal time and using cutting thicknesses identical to W0 and W16 for the measurement of CSA.

In conclusion, in patients with aPDAC receiving systemic chemotherapy, the loss of muscle mass measured by longitudinal analysis may influence OS and tend to be associated with HRQoL. The absence of a signal in favour in APA is consistent with the existing literature, but it may be possible to optimize both the APA intervention and longitudinal follow‐up, considering the complexity of their effect on muscle and the other biological determinants involved in muscle mass. Further assessments, including combination or more intensive nutritional and APA interventions with better tailored program, as well as the search for biomarkers and studies stratifying patients for multimodal interventions are warranted.

## Conflicts of Interest

The authors declare no conflicts of interest.

## Supporting information


**Appendix S1.** Supporting information

**Table S1.** Baseline patient characteristics in the ancillary SarcAPACaP study among arm of intervention
**Table S2.** Inter‐evaluators’ correlation on total muscle surface (A) and SMI status (B).
**Table S3.** Patient characteristics in the SarcAPACaP population according to the SMI status.
**Table S4.** Patient characteristics in the SarcAPACaP population according to the evolution muscle mass between the diagnosis and after 16 weeks.
**Table S5.** Relation between loss of weight and evolution of muscle mass
**Figure S1.** Time until definitive deterioration of physical functioning and fatigue in the mITT1 population and mITT2 population (A and B) and (C and D), respectively, according to SMI status.
