# Supplementary material for: Muscle Loss During First‐Line Chemotherapy Impairs Survival in Advanced Pancreatic Cancer Despite Adapted Physical Activity
Source: J Cachexia Sarcopenia Muscle. 2025 Jan 17;16(1):e13595. doi: 10.1002/jcsm.13595 (PMC11788588; doi:10.1002/jcsm.13595)
Supplement: Supplementary file 1 — Appendix S1. Supporting information [file JCSM-16-e13595-s001.docx]

**eAppendix 1. APACaP Study Protocol Information (cont.)**

**The 16-Week APA Program Exercises**

Aerobic exercises were performed with a moderate intensity (ie, that allowed the patient to speak comfortably). The muscle-strengthening program included repetitions performed at 50% of the baseline maximal strength test.

One-repetition maximum (1-RM) was a measure of muscular strength. It was the heaviest weight that could be lifted for one repetition and one repetition only.

Elastic bands (TheraBands) with 8 color-coded resistance level bands were frequently used to assess the 1-RM. The APA professional measured the distance from the starting position to the ending position during exercise with the elastic band to determine its deformation. After a warm-up without the elastic band the appropriate band was chosen for exercise based on the APA professional’s assessment of the patient’s approximate strength. If the patient could perform more than ten repetitions easily, a higher resistance band was tested. The amount of deformation, the color of

the elastic band, and the number of repetitions were required parameters to calculate the 1-RM. The 1-RM test was calculated based on the Brzycki equation: 1-RM5 weight loaded/(1,0278 – [0,0278 x number of repetitions]). The weight loaded was defined for each elastic band color depending on the level of deformation.

Physical activity sessions included warm-up exercises followed by aerobic or resistance exercises and a recuperation period. The warm-up and recuperation periods represented at least 30% of the session. The aim of the APA program was to gradually reach a total of 30 minutes of aerobic training, 3 to 5 times per week (depending on the physical condition), and to perform strengthening activities at least twice a week for all the major muscle groups, excluding the warm-up and recuperation periods. One week with lower intensity activity could be planned every 4 weeks to prevent patient exhaustion. Patients were asked to report on their APA program in a specific activity booklet. Photographs and explanations for good positioning and proper execution of exercises were provided to patients.
