# Supplementary material for: Muscle Loss During First‐Line Chemotherapy Impairs Survival in Advanced Pancreatic Cancer Despite Adapted Physical Activity
Source: J Cachexia Sarcopenia Muscle. 2025 Jan 17;16(1):e13595. doi: 10.1002/jcsm.13595 (PMC11788588; doi:10.1002/jcsm.13595)
Supplement: Supplementary file 2 — Table S1. Baseline patient characteristics in the ancillary SarcAPACaP study among arm of intervention Table S2. Inter‐evaluators’ correlation on total muscle surface (A) and SMI status (B). Table S3. Patient characteristics in the SarcAPACaP population according to the SMI status. Table S4. Patient characteristics in the SarcAPACaP population according to the evolution muscle mass between the diagnosis and after 16 weeks. Table S5. Relation between loss of weight and evolution of muscle mass Figure S1. Time until definitive deterioration of physical functioning and fatigue in the mITT1 population and mITT2 population (A and B) and (C and D), respectively, according to SMI status. [file JCSM-16-e13595-s002.docx]

**Supplementary Table 2.** Inter-evaluators’ correlation on total muscle surface (A) and SMI status (B).

A

| **Total Muscle Surface Area** | | | | | | |
| --- | --- | --- | --- | --- | --- | --- |
|  | W0  mean (std) | Correlation | W16  mean (std) | Correlation | Relative difference between W16 and W0 (%) mean (std) | Correlation |
| Observer 1 | 136,15 (28,08) | 0,99751 | 129,85 (27,33) | 0,99651 | -4,22 (5,85) | 0,97725 |
| Observer 2 | 138,75 (28,22) |  | 133,41 (27,91) |  | -3,51 (8,29) |  |
| Mean difference | -2,6*(1,99) |  | -3,56 (2,38) |  | -0,71 (1,82) |  |

B

| **SMI Status** |  | | | | | | | |
| --- | --- | --- | --- | --- | --- | --- | --- | --- |
|  |  | Observer 2 | | | | |  | |
|  |  | Normal SMI at W0  N=16 | | Low SMI at W0  N=12 | | | | Kappa (95%CI) |
|  |  | n | % | n | | % | |  |
| Observer 1 | Normal SMI at W0 | 14 | 87,5 | 0 | 0 | | | 0,8571 (0,67-1,0) |
|  | Low SMI at W0 | 2 | 12,5 | 12 | 100 | | |  |
|  |  | Normal SMI at W16 | | Low SMI at W16 | | | |  |
|  |  | N=12 | | N=16 | | | |  |
|  | Normal SMI at W16 | 11 | 91,67 | 0 | 0 | | | 0,9263 (0,78-1,0) |
|  | Low at W16 | 1 | 8,33 | 16 | 100 | | |  |

**Supplementary Table 3.** Patient characteristics in the SarcAPACaP population according to the SMI status.

|  | **mITT1 W0 population N=225(%)** | **Normal SMI patients N=122 (%)** | **Low SMI patients N=103 (%)** | **P-value** |
| --- | --- | --- | --- | --- |
| **ECOS PS** | | | | |
| 0-1 | 211 (94) | 116 (95) | 95 (92) | 0.378 |
| 2 | 14 (6) | 6 (5) | 8 (8) |  |
| **GPAQ** | | | | |
| Very active | 81 (36) | 48 (39) | 33 (32) | 0.517 |
| Moderately active | 71 (32) | 36 (30) | 35 (34) |  |
| Inactive | 73 (32) | 1038 (31) | 35 (34) |  |
| **Chemotherapy regimen** | | | | |
| Weekly | 25 (11) | 14 (11) | 11 (11) | 0.850 |
| Every 2 weeks | 200 (89) | 108 (89) | 92 (90) |  |
| **Age,** years, median (q1-q3) | 64 (56-70) | 62 (56-68) | 66 (58-72) | **0.005** |
| **Sex** | | | | |
| Male | 132 (59) | 88 (72) | 44 (43) | **<0.001** |
| Female | 93 (41) | 34 (28) | 59 (57) |  |
| **Tumor stage at inclusion** | | | | |
| Locally advanced | 52 (23) | 29 (24) | 23 (22) | 0.798 |
| Metastatic | 173 (77) | 93 (76) | 80 (78) |  |
| **Surgery of pancreatic tumor** | | | | |
| No | 205 (92) | 110 (90) | 95 (93) | 0.426 |
| Yes | 19 (9) | 12 (10) | 7 (7) |  |
| Unknown | 1 | 0 (0) | 1 (0) |  |
| **Number of metastatic sites** | | | | |
| 0 | 52 (23) | 29 (24) | 23 (22) | 0.926 |
| 1 | 117 (52) | 62 (51) | 55 (53) |  |
| ≥ 2 | 56 (25) | 31 (25) | 25 (24) |  |
| **BMI**, kg/m², median (q1-q3) | 23,6 (21,3-26,3) | 23,45 (21,6-26,2) | 23,8 (20,8-26,4) | 0,3888 |
| **Treatment arm** | | | | |
| Standard arm | 109 (48) | 51 (42) | 58 (56) | **0.03** |
| APA arm | 116 (52) | 71 (58) | 45 (44) |  |

Abbreviations: ECOG PS, performance status; BMI, body mass index; GPAQ, Global Physical Activity Questionnaire; APA, adapted physical activity.

**Supplementary Table 4.** Patient characteristics in the SarcAPACaP population according to the evolution muscle mass between the diagnosis and after 16 weeks.

|  | **mITT2 W0-W16 population**  ***N* = 128**  ***n* (%)** | **Loss > 10% *n* = 27**  ***n* (%)** | **Loss between**  **0 and 10%  *n* = 63**  ***n* (%)** | **No loss  N=38**  ***n* (%)** | **P-value** |
| --- | --- | --- | --- | --- | --- |
| **ECOS PS** | | | | | |
| 0-1 | 123 (96) | 25 (93) | 62 (98) | 36 (95) | 0.293 |
| 2 | 5 (4) | 2 (7) | 1 (2) | 2 (5) |  |
| **GPAQ** | | | | | |
| Very active | 44 (34) | 12 (44) | 22 (35) | 10 (26) | 0.132 |
| Moderately active | 45 (35) | 5 (19) | 21 (33) | 19 (50) |  |
| Inactive | 39 (31) | 10 (37) | 20 (32) | 9 (24) |  |
| **Chemotherapy regimen** | | | | | |
| Weekly | 11 (9) | 1 (4) | 6 (10) | 4 (11) | 0.658 |
| Every 2 weeks | 117 (91) | 26 (96) | 57 (90) | 34 (89) |  |
| **Age** mean (std) | 62.29 (11.32) | 60.59 (11.66) | 62.84 (11.72) | 62.58 (10.56) | 0.698 |
| **Sex** | | | | | |
| Male | 76 (59) | 23 (85) | 31 (49) | 22 (58) | **0.006** |
| Female | 52 (41) | 4 (15) | 32 (51) | 16 (42) |  |
| **Tumor stage at inclusion** | | | | | |
| Locally advanced | 28 (22) | 3 (11) | 14 (22) | 11 (29) | 0.229 |
| Metastatic | 100 (78) | 24 (89) | 49 (78) | 27 (71) |  |
| **Surgery of pancreatic tumor** | | | | | |
| No | 116 (91) | 25 (93) | 53 (85) | 38 (100) | **0.027** |
| Yes | 11 (9) | 2 (7) | 9 (15) | 0 (0) |  |
| Unknown | 1 | 0 (0) | 1 (0) | 0 (0) |  |
| **Number of metastatic sites** | | | | | |
| 0 | 28 (22) | 3 (11) | 14 (22) | 11 (29) | 0.546 |
| 1 | 69 (54) | 17 | 33 (52) | 19 (50) |  |
| ≥ 2 | 31 (24) | 7 | 16 (25) | 8 (21) |  |
| **BMI**, kg/m², median | 24.2 (16.3-35.5) | 22.9 (16.3-35.5) | 24.8 (17.3-34.6) | 22.65 (17.7-31.6) | 0.267 |
| **Treatment arm** | | | | | |
| Standard arm | 63 (49) | 13 (48) | 32 (51) | 18 (47) | 0.939 |
| APA arm | 65 (51) | 14 (52) | 31 (49) | 20 (53) |  |
| **SMI,** cm²/m², mean (std) | 45.68 (8.21) | 49.04 (7.48) | 45.49 (9.19) | 43,61 (6.15) | **0.039** |

Abbreviations: ECOG PS, performance status; BMI, body mass index; GPAQ, Global Physical Activity Questionnaire; APA, adapted physical activity; SMI, skeletal muscle index.

**Supplementary Table 5 :** Relation between loss of weight and evolution of muscle mass

| Body weight between W0 and W16 | No loss | 0-10% | > 10% | P value |
| --- | --- | --- | --- | --- |
| ≤5% | 26 (78.79) | 28 (45.90) | 2 (8.00) | 0,0001 |
| 5-10% | 3 (9.09) | 21 (34.43) | 6 (24.00) |  |
| >10% | 4 (12.12) | 12 (19.67) | 17 (68.00) |  |


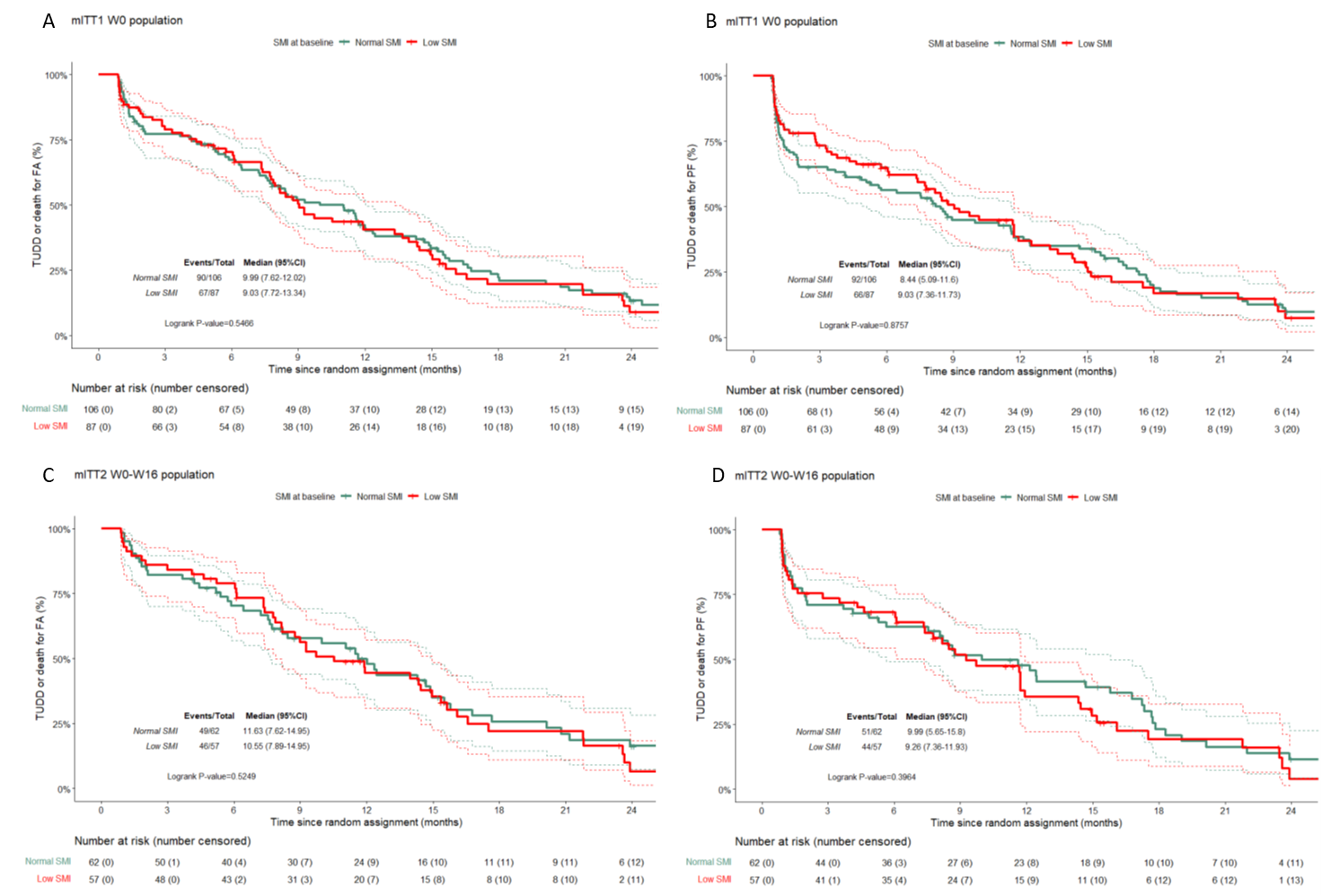


Supplementary Figure S1 Time until definitive deterioration of physical functioning and fatigue in the mITT1 population and mITT2 population (A and B) and (C and D), respectively, according to SMI status.
